# CTX-M-1 and CTX-M-15-producing *Escherichia coli* in dog faeces from public gardens

**DOI:** 10.1186/s13028-015-0174-3

**Published:** 2015-11-25

**Authors:** Peter Damborg, Malene Kjelin Morsing, Tanja Petersen, Valeria Bortolaia, Luca Guardabassi

**Affiliations:** Department of Veterinary Disease Biology, Faculty of Health and Medical Sciences, University of Copenhagen, Stigbøjlen 4, 1870 Frederiksberg, Denmark; Department of Biomedical Sciences, Ross University School of Veterinary Medicine, Basseterre, St Kitts West Indies

**Keywords:** ESBL, Canine, Environment, Antibiotic resistance, Zoonoses, CTX-M

## Abstract

**Background:**

Extended-spectrum beta-lactamase (ESBL)-producing *Escherichia coli* are increasingly reported in dogs. The objective of this study was to provide data on the prevalence of ESBL-producing *E. coli* in dog faecal deposits in public gardens.

**Results:**

A total of 209 faecal deposits collected in nine public gardens in Copenhagen, Denmark were screened by selective enrichment followed by plating on MacConkey agar supplemented with cefotaxime. Presumptive ESBL-producing *E. coli* were confirmed by MALDI-TOF MS and polymerase chain reaction (PCR) for detection of common cefotaxime resistance determinants (*bla*_TEM_, *bla*_SHV_, *bla*_CTX-M_ and *bla*_CMY-2_). ESBL-producers were further characterized by multilocus sequence typing (MLST) and antimicrobial susceptibility testing using broth microdilution. Plasmids harbouring ESBL genes were characterized by S1 nuclease pulsed field gel electrophoresis, PCR-based replicon typing, and pMLST. Cefotaxime-resistant *E. coli* were detected in four (1.9 %) samples. Three samples harboured CTX-M-1-producing isolates, and one sample contained two CTX-M-15-producing isolates displaying distinct colony morphology. All isolates belonged to distinct sequence types (STs), including one *E. coli* lineage previously associated to a human-specific pathotype (ST59). *bla*_CTX-M-1_ was carried on IncI1 plasmids classified as ST3 or ST58 by pMLST, whereas *bla*_CTX-M-15_ was located on IncF/Y and non-typeable plasmids in the two strains isolated from the same sample.

**Conclusions:**

The study shows that dog faeces are a vector for dissemination of CTX-M-producing *E. coli* within urban areas. The risk derived from human exposure to dog faeces in public gardens depends on the prevalence of these bacteria in the local dog population as well as on the owners’ practice to remove and dispose their dog’s faeces.

## Findings

The rapid emergence of extended-spectrum beta-lactamase (ESBL)-producing *Escherichia coli* in the community has prompted search for sources in the animal population. Food animals are a well-studied animal reservoir, but companion animals, in particular dogs, may also be colonised or infected with these cephalosporin-resistant *E. coli* [1]. The prevalence of ESBL carriage in dogs varies considerably between countries with the highest frequency (55 %) being reported in diarrheic dogs in The Netherlands [2]. Faecal shedding of ESBL-producing *E. coli* by dogs was recently shown to be highly dynamic with the majority of dogs being intermittent shedders and positive for different ESBL genes over time [3]. The most common ESBL type found in canine *E. coli* is CTX-M, which also predominates in humans and other animals including livestock and wild animals [1, 4, 5].

The close contact between dogs and humans implies risks of zoonotic transmission for a variety of pathogens, especially by the faecal-oral route [6]. Dog faeces are a recognized source of zoonotic agents, including clinically relevant resistant bacteria that can be transmitted to humans through direct exposure or via contamination of domestic and public environments. The objective of this study was to provide data on the prevalence and types of ESBL-producing *E. coli* in dog faecal deposits in public gardens.

In April and May 2013, 209 faecal samples were collected from trashcans in nine public gardens (5–40 samples per park) in the Greater Copenhagen area, Denmark. All samples were contained in plastic bags and supposedly less than 2 days old, since trashcans in these gardens are emptied at least every other day by the municipality. Each garden was visited only once to limit the risk of getting more than one sample per dog.

Samples were processed in the laboratory on the day of collection. One gram of each sample was suspended 1:10 in MacConkey broth (Oxoid, Basingstoke, UK) supplemented with 1 mg/l cefotaxime. Following overnight incubation with shaking at 37 °C, 10 µl of the enrichment culture was plated onto MacConkey agar (Oxoid) containing 1 mg/l cefotaxime. After incubation (24 h, 37 °C), one presumptive *E. coli* was sub-cultured from samples displaying growth and stored at −80 °C prior to further analyses. Multiple colonies were stored if more than one colony morphology was visible on the same plate.

Presumptive *E. coli* isolates were confirmed by matrix-assisted laser desorption ionization-time of flight (MALDI-TOF) mass spectrometry (Vitek MS RUO; bioMérieux, France) using *E. coli* ATCC 8739 as reference strain and Saramis™ 3.5 (bioMérieux) for spectra interpretation. Antibiotic susceptibility was tested by broth microdilution using Sensititre COMPAN1F plates (Thermo Fisher Scientific, Hvidovre, Denmark). Susceptibility to amoxicillin clavulanate, cefoxitin, cefpodoxime, imipenem, and the non-beta-lactams amikacin, chloramphenicol, doxycycline, enrofloxacin, gentamicin, marbofloxacin, and sulfamethoxazole/trimethoprim was interpreted according to the Clinical and Laboratory Standards Institute guidelines [7].

Genes encoding the most common ESBL types (*bla*_CTX-M_, *bla*_TEM_ and *bla*_SHV_) and plasmid-encoded AmpC beta-lactamase (*bla*_CMY-2_) were detected by polymerase chain reaction (PCR) and sequenced [8, 9]. Positive isolates were characterized by multilocus sequence typing (MLST) and assigned to sequence types (STs) using the *E. coli* MLST database (http://mlst-warwick.ac.uk).

Plasmid DNA purified by alkaline lysis was transformed into electrocompetent Genehog *E. coli* (Invitrogen, Carlsbad, USA) followed by selection of transformants on Müller Hinton agar (Oxoid, Basingstoke, UK) supplemented with 1 mg/l cefotaxime. Transformants verified by colony PCR were subjected to S1 nuclease pulsed field gel electrophoresis (PFGE) [10], PCR-based replicon typing (PBRT) (Diatheva, Fano, Italy), and antimicrobial susceptibility testing (as described above). IncI1 plasmids were subtyped by plasmid MLST (pMLST) [11].

Four faecal deposits (1.9 %) collected in two gardens displayed growth of lactose-positive colonies, and additional growth of lactose-negative colonies was detected in one of these samples. All colonies were identified as *E. coli* and harboured one or more genes encoding β-lactamase, namely *bla*_CTX-M-1_ (n = 3), *bla*_CTX-M-15_ (n = 2) and *bla*_TEM-1_ (n = 2) (Table 1). Each isolate belonged to a distinct genotype (ST59, ST609, ST4304, ST4305 or ST4340). The five strains were resistant to cefpodoxime and susceptible to amoxicillin clavulanate, cefoxitin, aminoglycosides and chloramphenicol. Susceptibility to fluoroquinolones, sulfamethoxazole/trimethoprim and doxycycline varied depending on the strain (Table 1).

**Table 1:** Genotypic and phenotypic features of cefotaxime-resistant *Escherichia coli* isolated from dog faecal deposits

Strain ID	Public garden	Faecal deposit	Cefotaxime-resistant *E. coli*			CTX-M-encoding plasmid			
			*bla* gene(s)	Resistance to non-beta-lactams	MLST type	Replicon(s)	pMLST type	Size (kb)	Co-transferred resistance
147	A	1	*bla*_CTX-M-1_	None	ST59	I1	ST58	78	None
115	B	2	*bla*_CTX-M-1_	SXT	ST4340	I1	ST58	78	None
122	B	3	*bla*_CTX-M-1_ *bla*_TEM-1_	DOX, SXT	ST4304	I1	ST3	104	SXT
104g^a^	B	4	*bla*_CTX-M-15_	ENR, MAR	ST4305	Non-typeable	NA	104	None
104r^b^	B	4	*bla*_CTX-M-15_ *bla*_TEM-1_	DOX, SXT	ST609	FIB, Y	NA	95	SXT

*ENR* enrofloxacin, *DOX* doxycycline, *MAR* marbofloxacin, *SXT* sulfamethoxazole/trimethoprim, *NA* not applicable

^a^Lactose-negative strain

^b^Lactose-positive strain

Plasmids carrying ESBL genes varied from approximately 78 to 104 Kb. *bla*_CTX-M-1_ was found in three strains on IncI1 plasmids assigned to ST58 (n = 2) or ST3 (n = 1) by pMLST. *bla*_CTX-M-15_ was detected in the lactose-positive and the lactose-negative isolates from the same sample on distinct plasmids, one positive for both the FIB and the Y replicon types, and the other one non-typeable by PBRT. Resistance to sulfamethoxazole and trimethoprim was co-transferred by the two plasmids IncI1 ST3 and FIB/Y (Table 1).

The observed prevalence of 1.9 % ESBL-producing *E. coli* in dog faecal deposits is generally lower than reported in healthy dogs in other European studies [2, 12, 13]. This difference may indicate a low prevalence of ESBL-producing *E. coli* in the Danish dog population but could also be due to differences in study population, sample collection or processing. In fact, most other studies reported on fresh samples obtained directly from dogs. The high local variation with most ESBL-positive samples deriving from public garden B (Table 1) may reflect the relatively high number of samples (n = 40) collected in this garden, but otherwise is difficult to explain given the close proximity of the gardens (within a 7 km diameter zone) and the common municipal practice for emptying of waste bins. One recent study detected ESBL or AmpC-producing *E. coli* in 14 % of faecal deposits collected near a small animal hospital in Germany [14]. This high prevalence was likely influenced by the study location and associated antimicrobial selective pressure, since some clones were also detected in hospitalized dogs. Both that and the present study found a clear predominance of *bla*_CTX-M-1_ and *bla*_CTX-M-15_ among isolates, thus confirming previous evidence that these ESBL-types are the most frequent in dogs in Europe [1, 3, 15]. The ESBLs were found in different *E. coli* genetic backgrounds, including three newly described STs (ST4304, ST4305 and ST4340) and one genotype (ST59) that has previously been associated to a human-specific pathotype [16]. Our failure to detect CMY-2 was however unexpected, as this plasmid-mediated AmpC β-lactamase is frequent among cephalosporin-resistant *E. coli* isolated from dogs in Denmark [17] as well as in other EU countries [18] and continents [19–21].

The association of *bla*_CTX-M-1_ with IncI1 plasmids is well known in *E. coli* from humans and several animal species across Europe. Within this incompatibility group, ST3 plasmids harbouring *bla*_CTX-M-1_ have been reported in different animal species in Denmark, France, Switzerland and Tunisia [13, 22–25], suggesting that this specific IncI1 plasmid lineage has no geographic or host barriers. The other IncI1 plasmid detected in association with *bla*_CTX-M-1_ (ST58) plasmids is less frequently reported but was recently described in human CTX-M-1-producing *E. coli* isolates from Danish patients [24].

CTX-M-15 is the most frequent ESBL type in human clinical *E. coli* isolates in most EU countries, often in association with IncF plasmids [4]. We found *bla*_CTX-M-15_ on IncFIB/IncY and non-typeable plasmids in two genetically distinct *E. coli* strains originating from the same sample (Table 1). Multireplicon plasmid IncFIB/IncY have not been reported previously, although another hybrid replicon (IncY/IncA/C) was described in association with *bla*_CTX-M-15_ in *Salmonella* Concord isolated from humans in France [26]. Our results suggest that the dog from which the sample derived had been exposed to CTX-M-15-producing *E. coli* on at least two occasions or that recombination took place between different plasmids and strains. This second hypothesis is supported by the fact that the IncY replicon is also found on phages, and several plasmids non-typeable by PBRT in *E. coli* are indeed phages [27].

A limitation of the study is that samples were inside plastic bags upon collection and therefore not fully representing the faecal deposits that people may encounter on the ground. However, we deliberately chose this sampling strategy to minimize the risk of collecting samples from the same dog more than once.

The study shows that dog faeces are a vector for dissemination of CTX-M-producing *E. coli* within urban areas. The risk derived from human exposure to dog faeces in public gardens is highlighted by the isolation of *E. coli* and plasmid lineages previously detected in human infections. This risk appears to be low in Copenhagen but could be significantly higher in other geographical areas where these bacteria are prevalent in the dog population and the practice of removing and disposing dog faeces is not well established among dog owners. In view of this and the potential for other zoonotic pathogens in dog faeces [6], owners should be encouraged to dispose dog faecal deposits and to practice hand hygienic procedures afterwards, for example by using a hand rub disinfectant.

## Abbreviations

ESBL: extended-spectrum beta-lactamase
MALDI-TOF MS: matrix-assisted laser desorption ionization-time of flight mass spectrometry
MLST: multilocus sequence typing
PFGE: pulsed field gel electrophoresis
PBRT: PCR-based replicon typing
pMLST: plasmid multilocus sequence typing
ST: sequence type
